# 10,600 nm High Level-Laser Therapy Dosimetry in Management of Unresponsive Persistent Peripheral Giant Cell Granuloma to Standard Surgical Approach: A Case Report with 6-Month Follow-Up

**DOI:** 10.3390/jpm14010026

**Published:** 2023-12-25

**Authors:** Reem Hanna, Stefano Benedicenti

**Affiliations:** 1 Department of Restorative Dental Sciences, UCL-Eastman Dental Institute, Medical College, University College London, London WC1E 6DE, UK; 2 Department of Surgical Sciences and Integrated Diagnostic, University of Genoa, 16132 Genoa, Italy; stefano.benedicenti@unige.it; 3 Department of Oral Surgery, King’s College Hospital, London SE5 9RS, UK

**Keywords:** carbon dioxide laser, *λ* 10,600 nm, giant cell granuloma, HLLT, pain, peripheral giant cell granuloma, wound healing, lesion resolution, photothermal, protein heat shock

## Abstract

Peripheral giant cell granuloma (PGCG) is a non-neoplastic, tumour-like reactive lesion that exclusively involves the gingiva and/or the alveolar crest. The surgical approach with a scalpel has been the golden standard of treatment for PGCG, but the scientific literature reports a high rate of lesion recurrence. Hence, this unique case report aimed to evaluate the efficacy of *λ* 10,600 nm high-level laser therapy (HLLT) in eradicating persistent, aggressive, and recurrent PGCG that failed to respond to standard surgical treatment. A fit and healthy thirty-four-year-old Caucasian male presented with a two-month history of recurrent episodes of an oral mucosal lesion involving the buccal and lingual interdental papillae between the lower right second premolar (LR5) and lower right first molar (LR6), which was surgically excised with a scalpel three times previously. A *λ* 10,600 nm-induced HLLT was chosen as a treatment modality at a lower peak power of 1.62 W, measured with a power metre, emitted in gated emission mode (50% duty cycle), whereby the average output power reaching the target tissue was 0.81 W. The spot size was 0.8 mm. Ninety seconds was the total treatment duration, and the total energy density was 7934.78 J/cm^2^. Patient self-reporting outcomes revealed minimal to no post-operative complications. Initial healing was observed on the 4th day of the post-laser treatment, and a complete healing occurred at two-weeks post-operatively. The histological analysis revealed PGCG. This unique case report study demonstrated the efficacy of *λ* 10,600 nm-induced HLLT and its superiority to eradicate persistent aggressive PGCG over the standard surgical approach with minimal to no post-operative complications, accelerating wound healing beyond the physiological healing time associated with no evidence of PGCG recurrence at the six-month follow-up timepoint. Based on the significant findings of this unique study and the results of our previous clinical studies, we can confirm the validity and effectiveness of our standardised *λ* 10,600 nm laser dosimetry-induced HLLT and treatment protocol in achieving optimal outcomes. Randomised controlled clinical trials with large data comparing *λ* 10,600 nm with our dosimetry protocol to the standard surgical treatment modality at long follow-up timepoints are warranted.

## 1. Introduction

Peripheral giant cell granuloma (PGCG) is a non-neoplastic, tumour-like reactive lesion occurring exclusively in the gingiva or and alveolar crest. It appears that it is erupted from the periodontal ligament or the periosteum [1]. Giant cell granulomas occurring within the bone are of central origin and called “central giant cell granuloma” (CGCG). However, those lesions occurring on the edentulous alveolar processes or on the gingivae are PGCG. Although the CGCG is rare in nature, it makes up 7% of the total benign jaw lesions, especially in young patients [2,3]. Contrary to this, PGCG is a more common giant cell lesion of the jaw, arising in response to a local irritation from the gingival connective tissue or periodontal membrane or from the periosteum of the alveolar ridge [4]. In terms of the demographic characteristics of PGCG, it does not have an age predilection, but it appears to have a greater predilection in females and tends to occur more frequently in the molar region [5,6,7]. The clinical features of PGCG are essentially identical to those of CGCG and intrabony benign neoplasm of the jawbone, but its unique histomorphology characteristic can differentiate it from CGCG [8].

### PGCG Excision with Surgical Lasers vs. Conventional Surgical Approach

Recurrent episodes of PGCG with conventional treatment modalities such as scalpel are attributed to a lack of deep excision to include the periodontal ligament [9], which can be related to the surgical technique [9,10,11], whereby re-excision ought to be performed [12]. PGCG lesions are normally self-limited [13], but the lesion in our case report study was persistent and required excision on multiple occasions with rapid eruption of the lesion within two weeks of its excision.

Interestingly, a literature review of 2824 PGCG lesions conducted by Chrcanovic et al., (2018) [9] reported an overall recurrence rate of 9.5% for PGCGs after treatment, but it was 16% if the lesion was excised with only conventional treatment modalities. However, if additional bony curettage was performed, the recurrence rate would drop to 2.8%.

It is noteworthy that aggressive tendencies or malignant transformations of PGCG have never been reported in the current literature [14,15]. Nevertheless, in our case, the lesion was aggressive in nature with a rapid and high recurrence rate; hence, it is fundamental to control the disease progression by reducing the pathogen via a thorough decontamination of the affected region. Hence, high-level laser therapy (HLLT) can be considered as an alternative to the standard surgical treatment modality.

Several clinical studies have employed surgical laser (HLLT), including diodes [16,17,18,19], carbon dioxide laser (CO_2_) [20,21,22,23,24], and the Erbium family (Er:YAG and Er,Cr:YSGG) [25,26,27,28], for a variety of indications due to their bactericidal and regenerative properties [29], but the evidence is scant in relation to PGCG management [17,28] especially for the persistent and rapid eruption type of lesion. Moreover, in terms of the complete healing time, there is a debate in the literature between conventional treatment modalities and surgical laser treatments of various wavelengths (HLLT). Nevertheless, the evidence is very clear and well documented in the scientific literature; surgical wounds excised with 10,600 nm have fewer myofibroblasts compared to the scalpel wounds [30,31]. However, *λ* 10,600 nm laser wounds display less contractility than the scalpel wounds [30,32], resulting in less scarring. A prospective clinical study with large data conducted by Hanna et al., (2016) [33] demonstrated a complete healing of the oral mucosal tissues excised with *λ* 10,600 nm-induced HLLT at two weeks post-operatively with no evidence of scarring. This was in agreement with another case report study, whereby *λ* 10,600 nm was utilised in the management of denture-induced hyperplasia and vestibuloplasty in a medically compromised patient [34].

HLLT-mediated reaction of surgical wavelengths such as *λ* 2780 nm, *λ* 940 nm, or *λ* 10,600 nm results in tissue ablation with a peripheral zone of simultaneous low-level laser therapy (LLLT) around the surgical site, which is lacking in conventional treatment modalities [35,36]. HLLT is defined as high levels of incident laser power that are utilised to instigate the photodestruction of a specific target tissue through a light–heat transduction process, inducing photothermal damage of varying degrees [36].

**Figure 1:**
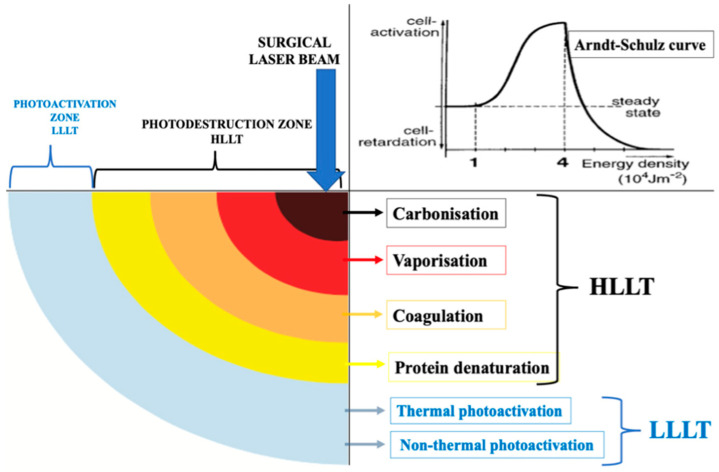
A schematic illustration of the concept of the surgical laser beam profile where the photonic energy of *λ* 10,600 nm at its first zone (HLLT) is absorbed by water, which is subsequently transformed into thermal energy and then causes tissue destruction (tissue ablation), whereas the last zone of laser beam is LLLT (modified Hanna et al., 2022, permission obtained [37]). The top right corner of the graph is an “Arndt–Schulz curve”, illustrating the biphasic dose response measured in the difference in the integrated area under the curve of the time course of the wound size compared to a no-treatment control with different modes of cell reaction at different levels of energy density [38].

Figure 1 schematically illustrates the simultaneous low-level laser therapy (LLLT) concept, whereby an area of thermal and nonthermal photoactivation is produced simultaneously at the periphery of a high-powered surgical laser beam (HLLT) together with photodestructive reactions [37]. Also, Figure 1 illustrates the Arndt–Schulz curve concept of the Gaussian beam profile of LLLT [38]. It is noteworthy that LLLT is the formal term for the current named therapy called “photobiomodulation (PBM)”. It is important to consider the significant influencing factors such as wavelength, laser dosimetry, treatment protocol, and tissue optical properties, as well as the nature of the target lesion, which ultimately play a crucial role in achieving optimal outcomes [39,40,41].

In lieu with the above-mentioned notes, our case report aimed to demonstrate the efficacy of HLLT-mediated 10,600 nm to eradicate persistent recurrent PGCG. The objectives were as follows: (1) to assess the ablation/resection quality of the lesion with minimal to no peri-operative and post-operative complications; (2) to evaluate the healing time; (3) to ensure complete resolution of the lesion without evidence recurrence at two-week and 6-month timepoints.

## 2. Materials and Methods

### 2.1. Study Design

An interventional clinical prospective case report study evaluated the efficacy of *λ* 10,600 nm in complete excision of a persistent PGCG lesion with six month follow-up to ensure no relapse, minimal to post-operative complications and rapid wound healing.

This case report was conducted in accordance with the Declaration of Helsinki, and the laser procedure was approved by the Department of Oral Surgery, King’s College Hospital NHS Foundation Committee. An informed written consent was obtained from the patient, confirming a full understanding the proposed laser treatment, benefits, advantages, drawbacks, and alternative treatments. Additionally, an informed written consent was obtained from the patient for scientific and photos publications.

### 2.2. Study Participant and Case Description

A fit and healthy 34-year-old Caucasian male was referred by his general dental practitioner to the Oral Surgery Department, King’s College Hospital NHS Foundation Trust, London, UK, regarding a persistent recurrent intraoral lesion that occupied the interdental region between the lower right second premoral tooth (LR5) and lower right first molar (LR6). It was previously excised surgically with a scalpel three times without resolution. The time interval between the 3rd episode of lesion excision and the 4th episode of its recurrence was within two-weeks, when the patient presented to our clinic. The lesion was asymptomatic, but the patient experienced mild discomfort during brushing the mentioned teeth. 

Although the patient meticulously maintained a good oral hygiene at home and through regular hygienist appointments, the lesion recurrence was rapid and aggressive. The patient was a non-smoker and never smoked. He was not on any medications and without any history of any malignancies or allergies or any systematic diseases.

#### 2.2.1. Clinical Examination

**Figure 2:**
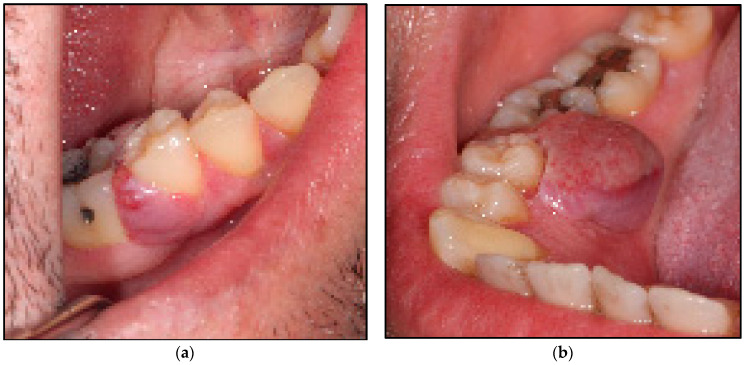
Shows the clinical presentation of the oral mucosal lesions. (**a**) represents erythromatous, pedunculated lesion with irregular texture, moderate in consistency, about 1 × 1 cm in diameter occupying the buccal gingivae and the entire interdental region between LR5 and LR6 buccally, firmly attached to the underlining structures; (**b**) represents a lobulated erythromatous, spongy (moderate in consistency) lesion about 1 × 1.5 × 2 cm in size, occupying the entire interdental papillae and the lingual mucosa between LR5 and LR6, firmly attached to the underlining structures.

Extraoral examination revealed no pathological abnormalities including no facial asymmetry or no lymphadenopathy. Whereas, intraoral examination revealed an exophytic granulomatous lesion associated with the buccal marginal gingivae of the LR5 and LR6, invading the interdental papillae to loop lingually and extended to involve the lingual marginal gingivae of the above-mentioned teeth. The lesion was an erythromatous, sessile or pedunculated nodule with an irregular texture that was elastic moderate in consistency, an isolated lump of 1 × 1 cm in diameter on the buccal aspects of LR5 and LR6 (Figure 2a) and 1 × 1 × 1.5 cm in diameter on the lingual surfaces fixed to the underlying structures of the above-mentioned teeth (Figure 2b). There was no sign of induration or ulceration, and it tended to bleed on probing and was pulsatile on palpation. The rest of the oral mucosal tissues were normal. All the teeth were immobile clinically and there was no evidence of any dental abnormalities.

#### 2.2.2. Investigations

**Figure 3:**
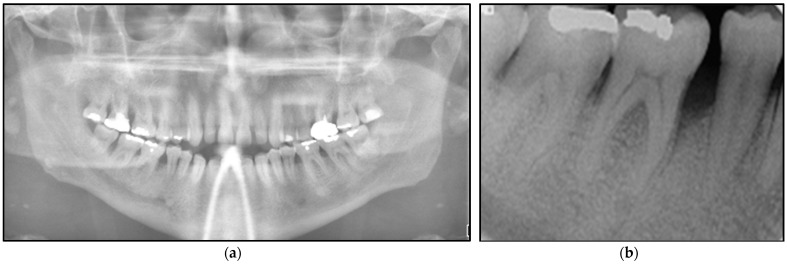
Shows the radiographic investigations. (**a**) represents the orthopantomogram imaging (extraoral) was taken in order to determine and evaluate the level of bone loss in the area of lower right 2nd premolar (LR5) and 1st molar (LR6) where the intraoral lesions presented compared with the contralateral side. It shows generalised bone loss without involving the bifurcation among the lower molars. (**b**) represents along-cone intraoral periapical view showing vertical bone loss distal to the LR5 and mesial to the LR6, whereby the intraoral lesion was presented intraorally. The evidence of bone loss is an indicative of the extension of the lesion beyond the soft tissue reaching the periosteum and alveolar bone. Otherwise, both images show no pathology.

Orthopantomogram (Figure 3a) and long-cone periapical views (Figure 3b) showed an evidence of a localised vertical bone loss involved LR5 distally and LR6 mesially, whereas the rest of the bone levels were within the normal range. It was indicative that the extensive of the lesion led to alveolar loss. 

All the following tests revealed within normal range: full complete blood picture, thyroid function test, liver function test, serum level of calcium and phosphorus.

#### 2.2.3. Differential Diagnosis

The differential diagnoses based on clinical and radiographical examinations were as follows: PGCG, pyogenic granuloma, haemangioma, CGCG or peripheral ossifying fibroma or metastatic carcinoma.

### 2.3. Interventions

#### Carbon Dioxide (*λ* 10,600 nm) Surgical Laser

As *λ* 10,600 nm is in the far end of the electromagnetic spectrum (EMS), it offers a shallow penetration depth of ~300 µ, and hence it gives the operator a great control of tissue ablation and also provides an excellent haemostatic effect [21,22], which is necessary in the management of this case. Additionally, this wavelength prompts anti-inflammatory and analgesic effects, favouring minimal to no post-operative complications. 

Moreover, in terms of the light property, light emerging from a light source can be altered before it is incident on the target tissue. It can be gathered and homogenised into a beam with a set of condensers, and the beam can be passed through a lens either to focus or to defocus it, even in the case of a conventional filament bulb, although the photon density is very small. In the case of a solid-state or gas-based laser such as the Nd:YAG or CO_2_, respectively, no condensing of the beam is required, because the laser energy emerges from the cavity in a beam which is already parallel or collimated [42].

The treatment options were as follows: surgical excision with a scalpel, cryosurgery, surgical laser treatment with diodes family; CO_2_ or Erbium family. Taking into consideration the lesion recurrent behaviour after being surgically excised with scalpel three times, it would be justifiable to consider an alternative method to ensure a complete eradication and resolution of the lesion.

In the lieu of the lesion recurrent history and behaviour, clinical and radiographic examinations, and the results of the blood work-up, the treatment rationale in this case was laser-assisted surgical excision. A *λ* 10,600 nm surgical laser was chosen, as it provides superior properties over the conventional surgical treatment in terms of reduction in pathogens and minimal to no post-operative complications [43,44,45]. Additionally, as the lesion was firmly attached to the LR5 and LR6 and deep-seated in the periosteum, a shallow penetration depth of the *λ* 10,600 nm (far end of the EMS) was ideal to provide the operator with the control in delivering the photonic energy, and hence it is the most suitable surgical wavelength compared with the diodes and Nd:YAG surgical wavelengths. Also, *λ* 10,600 nm is superior to the Erbium family in achieving optimal haemostasis perioperatively with shorter surgical time [46]. 

### 2.4. Dosimetry of λ 10,600 nm Surgical Laser 

**Figure 4:**
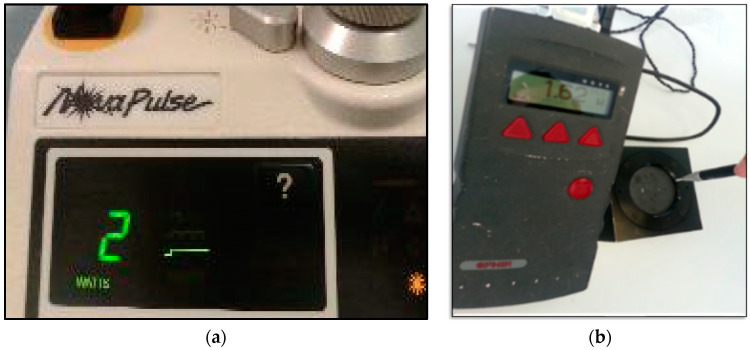
(**a**) Representation of the laser device panel showing 2 Watts power output, but when it was measured with the power meter (**b**), the power output exhibited on the power mater panel was 1.62 W (**b**). This indicates that there was a loss of 0.38 Watt in the fibre of the laser device.

The minimal output power provided on a Nova Pulse Lumenis panel is 2 Watts (W) (Figure 4a). We utilised a power meter (OPHIR laser power meter NOVA Display 7Z01500 + SENSOR FL500A7Z02648) to measure the therapeutic power output delivered to the target tissue, which was of value of 1.62 W shown in the power meter device (Figure 4b). 

As we are always aiming to utilise a minimal therapeutic output power delivering optimal outcome with minimal to no post-operative complications, a gated continuous emission mode (50% duty cycle) was employed to allow a thermal relaxation time. Hence, the average therapeutic output power was 0.81 W.

Clearly, there was 0.38 W of photonic energy loss in the fibre of the device. The standardised laser dosimetry protocol in the present study was based on our previous clinical scientific works [33,34,47]. Hence, it was employed for the management of aggressive and persistent PGCG. Table 1 shows the laser device specifications, laser dosimetry and treatment protocols.

### 2.5. Description of Laser-Assisted Surgical Approach

The following safety measures were implemented prior to the laser treatment: operating room secured with defined controlled area, laser warning signs illuminated; laser test fire checked and all the staff who were in the laser including the patient wore laser safety eyewear. All laser safety measurements were respected in accordance with the American National Standards Institute (ANSI) guideline [48].

The laser device was calibrated and tested prior to the treatment. The operator is an experienced surgeon and senior researcher with a wealth of experience in utilising HLLT surgical tools and PBM therapy.

Prior to the treatment, the patient confirmed their consent, and any questions were answered. Buccal and lingual infiltration of the local anaesthetic (LA) was administered (2% lignocaine in 1:80,000 adrenaline) in the region of LR5 and LR6, where only 0.8 mL of LA was needed to anaesthetise the region. 

The laser excision approach was from the base of the lesion buccally, and then the lingual part of the lesion was excised. The direction of the laser beam was parallel to the tooth structure and periosteum and perpendicular to the target tissue at a distance <1 mm away from the tissue. The harvested specimens were sent for histological examination, and it was written on the prescription that the lesion was excised with a CO_2_ laser. Additionally, after surgical laser excision, a deep curettage of the periosteum was performed using a curettage hand instrument to ensure no further daughter cells remained, which can contribute to the recurrence of the lesion [9]. The operator’s approach in utilising a hand instrument for deep periosteum curettage rather than a laser was a fundamental to avoid any laser light–bone interaction, knowing the predominate absorbent chromophore for 10,600 nm is water and not hydroxyapatite [33]. Haemostasis was achieved during and immediately after surgical laser excision.

In terms of minimising the collateral thermal effect, exogenous coolants were utilised in terms of an external water irrigation based on 12 mL/min flow, and a high-speed suction was utilised to remove the generated plume and provide air as a coolant. Additionally, our employed emission mode was based on a 50% duty cycle providing a thermal relaxation time, allowing heat dissipation. Also, a damp gauze was used to remove the desiccated char between each laser pass, ensuring effective laser excision. 

The patient was familiarised with the assessment tools, and post-operative instructions were explained and given (verbally and written). Review appointments were scheduled at four and fourteen days post-laser treatment as well as at six months post-operatively.

### 2.6. Laser Nurse Checklist of Variables One-Day Post-Operatively

All the following data were based on the patient self-reporting outcomes with scoring obtained via telephone call one day post-laser treatment by an independent laser nurse who was not involved in the study: pain intensity, bleeding, infection, swelling, analgesic usage (dose and frequency of paracetamol) and patient’s satisfaction. Patient-reported symptoms are very vital qualitative parameters after surgical treatment [49]. All the data were stored on a Microsoft Excel spreadsheet.

### 2.7. Outcome Assessment Measures

#### 2.7.1. Visual Analogue Scale (VAS)

The score of patient self-reporting pain was assessed using VAS, which consists of psychometric response scales to measure subjective response ranging “0–5”, representing “does not hurt” to “hurts the worse”, respectively [50,51]. This was assessed at one day after laser treatment via telephone call and on the 4th day after treatment at the clinic. Any analgesics taken including dosage were reported as well.

#### 2.7.2. Wound Healing

The Clinical Wound Healing Grading [52] is based on the following descriptive rating scale: Grade I: sloughy, Grade II: no granulation, Grade III: granulation, Grade IV: re-epithelialisation, Grade V: completely epithelialised. This was used to assess the healing time and the quality of wound healing on the 4th day and two weeks post-laser treatment at the clinic.

#### 2.7.3. Haemostasis Assessment Tool

The bleeding scoring tool [53] that was utilised to assess haemostasis was based on the following grading: 0: no bleeding; 1: oozing stop with applying pressure; 2: oozing, unresponsive to applied pressure and 3: bleeding. The bleeding was assessed peri-operatively, immediately after the laser treatment at the clinic and at one day after the treatment via telephone call.

#### 2.7.4. Inflammation, Oedema and Infection Assessment

This was assessed one-day after laser treatment via telephone call and on the 4th day of laser surgery at the clinic.

#### 2.7.5. Patient Satisfaction Assessment

The patient self-reported satisfaction scoring was based on a Modified Wong Baker Faces [33] scale on the following grades: 1: bad; 2: good; 3: very good; 4: excellent, evaluating patient’s treatment satisfaction immediately after treatment at the clinic, one day post-laser treatment via telephone call, on the 4th day of laser surgery at the clinic, and at a 8-month follow-up timepoint at the clinic.

## 3. Results

We evaluated all the outcomes of all the variables according to our study timepoints protocol, and the experimental results’ description and interpretation are outlined below.

### 3.1. Pain, Swelling, Infection and Patient’s Satisfaction

The patient self-reported mild pain “2” on VAS one day after the laser treatment via telephone call made by a laser specialist nurse who was not involved in the study. The patient also reported no need for analgesic intake. Then, the patient reported no pain “0” on VAS during the follow-up timepoints at the clinic.

In terms of infection and swelling, the patient self-reported one day post-treatment at home via telephone call was no post-operative complications, and this was applied to the following timepoints at the clinic.

The patient’s self-reporting score for treatment satisfaction was “3” immediately after treatment, one day after treatment and “4” at all the follow-up timepoints. At the telephone call appointment, the patient reported no inflammation or oedema or bleeding. All the data were obtained by a healthcare professional who was not involved in the study.

### 3.2. Haemostasis Assessment

**Figure 5:**
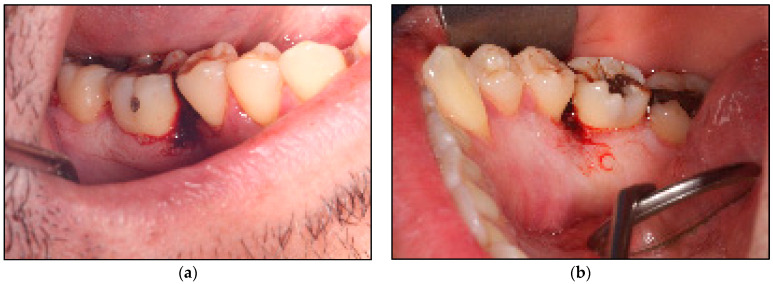
Clinical photos of the treated sites, (**a**,**b**), immediately after laser-assisted treatment, showing no evidence of oozing, and the clot is stable at the surgical sites.

The *λ* 10,600 nm surgical laser provided an excellent haemostasis properties peri and immediately post-laser treatment (Figure 5a,b). The scoring was “0” (no bleeding). Additionally, the patient did not report any bleeding or oozing one-day after surgery via telephone call review. 

### 3.3. Histopathological Interpretation

The findings showed an overlying epithelium had hyperplastic parakeratinised stratified squamous epithelium exhibiting areas of pseudoepitheliomatous hyperplasia. The proliferation of multinucleated giant cells and mixed inflammatory cells infiltration consisting predominantly of neutrophils, and lymphocytes were seen. Highly vascular connective tissue exhibiting numerous mononuclear stromal cells and extravasated red blood cells were evident. There was no evidence of atypia or any dysplastic tissue. Taking into account the histopathological findings and clinical features, the definitive diagnosis was ulcerated PGCG.

### 3.4. Wound Healing

**Figure 6:**
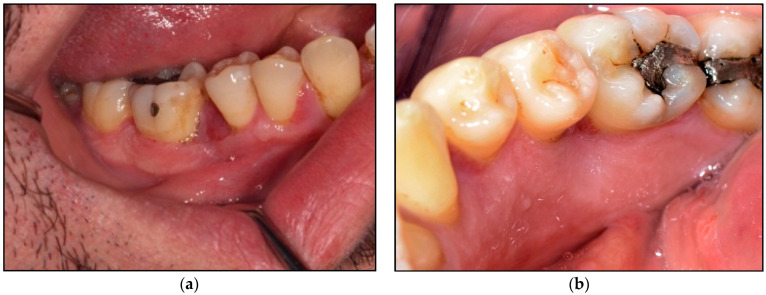
Clinical photos illustrating the outcomes of the laser treatment on the 4th day post-operatively. (**a**) shows Grade IV re-epithelisation of the mucosal tissue buccally as well as lingually (**b**) with well-maintained integrity of the gingival contouring and interdental papilla between LR5 and LR6.

In terms of wound healing, Figure 6 shows evidence of a band of re-epithelisation (Grade IV) of the mucosal tissues buccally (Figure 6a) and lingually (Figure 6b) on the 4th day post-laser treatment. Additionally, the clinical photos (Figure 6) show that the gingival contouring of LR5 and LR6 buccally and lingually was well maintained as well as the interdental papilla without evidence of gingival recession or lesion recurrence.

**Figure 7:**
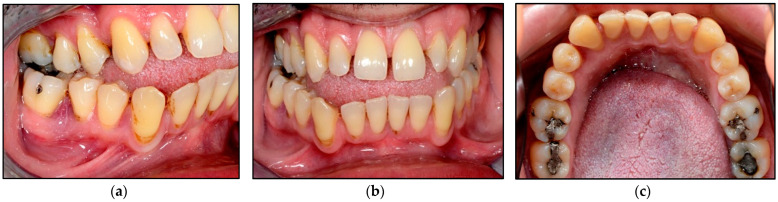
Shows clinical photos of the laser treated sites at two-weeks post-operatively. (**a**) an intraoral right lateral view of LR5 and LR6 demonstrates a complete re-epithelisation of the buccal mucosal tissue with no evidence of lesion recurrence or gingival recession. (**b**) an intraoral frontal view shows that the gingival zenith of the treated sites is aligned with the contralateral sites, demonstrating that laser-assisted surgery maintains the integrity of the gingival contour. (**c**) lingual view of the lower teeth shows a complete re-epithelisation of the lingual mucosal tissue of the treated sites (LR5 and LR6) with no evidence of lesion recurrence or gingival recession, as well the gingival zenith of the treated sites is aligned with the contralateral sites. This demonstrates that laser-assisted surgery does not jeopardise the integrity of the gingival contour.

At two weeks follow-up at the clinic, Figure 7a,c shows the treated sites exhibiting a complete epithelisation scoring Grade V with no evidence of lesion recurrence, and the gingival contouring around the LR5 and LR6 was healthy and pinkish in colour without evidence of gingival recession. The gingival zenith of the treated sites was aligned with the contralateral sites (Figure 7b,c). The evaluation of wound healing grading was performed by two healthcare professionals (the operator and the laser nurse) at all timepoints. The patient cancelled his 6-month follow-up appointment, but he confirmed no lesion reoccurrence via telephone call. 

## 4. Discussion

Although the scalpel or electrosurgery has been the first-line treatment choice for the surgical excision of most oral mucosal lesions, *λ* 10,600 nm-induced HLLT is becoming a widely accepted alternative treatment modality in the management of oral mucosal pathologies. 

The findings of the present study proved that *λ* 10,600 nm-induced HLLT is effective in eradicating long-standing, persistent, aggressive and rapidly growing PGCG lesions with minimal to no post-operative complications and achieving a complete resolution of the lesion confirmed on the 4th day and 14th days post-operatively at the clinic and at 6-months follow-up via telephone call. The utilisation of an appropriate wavelength, laser dosimetry and treatment protocols, understanding tissue optical properties and lesion nature, and operator’s surgical laser skills can collectively contribute to achieving optimal clinical outcomes with minimal post-operative complications. Hence, our results were significant in PGCG resolution without evidence of recurrence at the two-week follow-up timepoint, which was the critical timing for the lesion recurrence according to the patient’s records. Also, at 6-months follow-up, the patient reported, via telephone call, no further episodes of lesion recurrence.

**Figure 8:**
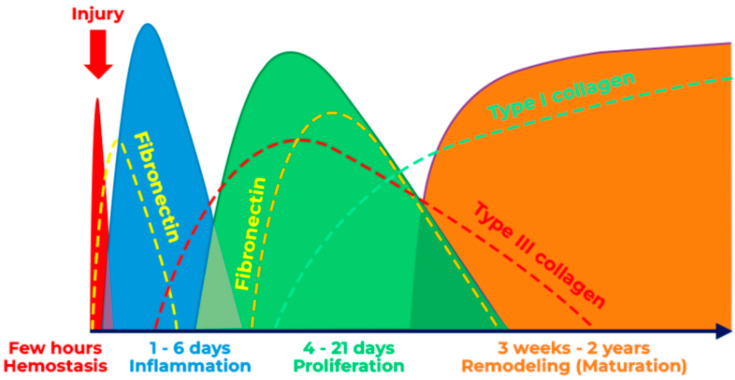
Shows the physiological wound-healing phases [Accessed 5 December 2023 via https://humanbiosciences.com/woundcareblog/what-is-wound-healing/].

Several studies have demonstrated the effectiveness of *λ* 10,600 nm in the management of various oral soft tissue conditions and pathologies with complete healing at two-weeks post-operatively [33,34]. The *λ* 10,600 nm laser properties provide a good tissue re-epithelialisation with minimal to no scar formation [30], and during the coagulation mode, it upregulates the expression of heat shock protein 25 (Hsp25), which is involved in the progress of wound repair [54]. It was evident in our study that re-epithelisation of the oral mucosal tissue (Grade IV) occurred at the 4th day post-laser treatment, indicating a “proliferation phase”, compared with the physiological wound healing of tissue excised with a standard treatment modality, which takes between 4 and 21 days (Figure 8) [55]. 

It is noteworthy that our findings demonstrated a complete healing of the wound at two weeks post-operatively (Grade V), indicating “maturation phase”, where the physiological healing time for normal wound healing at this phase is between three weeks and two years (Figure 8) [54]. Hence, our findings are in agreement with previous studies conducted by Hanna et al., (2016 and 2019) [33,34] utilising the same *λ* 10,600 nm dosimetry and treatment protocols, confirming its efficacy to accelerate wound healing compared with the standard treatments care (conventional surgical methods) and clearing the current scientific literature debate [30,31]. Hence, our standardised *λ* 10,600 nm dosimetry protocol can be a useful guide for clinicians utilising this wavelength for the surgical management of oral mucosal lesions and conditions. 

The significance of HLLT in providing a photobiomodulatory effect at the peripheral zone of the surgical site plays a crucial role in provoking analgesic properties [39,40,41], which ultimately offer pain relief post-laser treatment. It is noteworthy that PBM effects can also enhance wound healing and provide anti-inflammatory effects by upregulating cytokines reducing inflammation and oedema [56,57]. Thus, the *λ* 10,600 nm laser can be considered an excellent surgical tool in PGCG management, as tissue fluid is readily absorbing the photonic energy at the outer surface. Hence, only a little energy can be transmitted to the adjacent tissues [58] compared with the shorter wavelengths such as diodes [16,17,18,19], indicating minimal collateral thermal effects on the adjacent healthy tissues. Importantly, a high water absorption is inversely proportional to the reflection, scattering, and transmission of infrared radiation, which accounts for minimal dispersed energy and collateral thermal effects to the surrounding tissues not exceeding a range of 50 μm [59]. This advantageous property was very crucial in the present study, as obtaining a deep excision near the periosteum was a compulsory requirement to ensure a complete eradication of the PGCG lesion [19] without jeopardising the integrity of the adjacent healthy structures including the alveolar bone.

The implementation of minimal operating parameters and thermal relaxation can collectively reduce post-operative pain and accelerate healing time [33,34]. Hence, in the present study, we employed laser dosimetry protocol based on minimal output power and energy commensurate with optimal outcomes. We utilised an output power of 1.62 W measured with a power meter to determine the therapeutic power reaching the target tissue emitted in a 50% duty cycle, offering an average output power of 0.81 W and thermal relaxation time. This ultimately in conjunction with the advantageous properties of *λ* 10,600 nm and exogenous coolants (water and air) usage led to minimal to no post-operative complications and faster healing time beyond the physiological healing phase. On this note, a study conducted by Pié-Sánchez et al. (2012) [46] utilised 10,600 nm but at a higher peak power output of 5 W emitted in a continuous emission mode (CW) for upper midline frenectomy. They reported a complete healing time on the 21st day post-laser treatment. It is important to appreciate that in the present study, the complete healing time was at two weeks postoperatively. This demonstrates that employing a high-power output emitted in a CW with a lack of thermal relaxation time and exogenous coolant can fundamentally delay the healing time and contribute significantly to the collateral thermal damage, and ultimately, it can result in a negative impact on the integrity of the surrounding healthy tissues. 

Another advantageous property of *λ* 10,600 nm is a shallow penetration depth (~ 300 µm) with a minimal collateral thermal effect. This was potentially significant in our study, allowing the operator control to remove the lesion safely, as it was firmly attached to the periosteum and deep excision was required. Also, *λ* 10,600 nm (far end of EMS) provides an excellent haemostatic effect, allowing bloodless field throughout the excisional procedure and bleeding arrest immediately after the treatment and post-operatively. This ultimately related to the choice of *λ* 10,600 nm and therapeutic output power reaching the target, determining the penetration depth of the photonic energy and thus influencing the interplay between the tissue removal and haemostasis. Overall, the *λ* 10,600 nm optical absorption, light attenuation and coagulation depth are significantly greater than the blood vessel diameters, ranging from 21 to 40 μm, and hence the photocoagulation occurs over the extended volumes [21,22], offering a significant bloodless surgical field perioperatively and excellent haemostasis immediately after treatment.

Based on the strong evidence-based science and practice published in the literature, the efficacy, validity and safety of our standardised *λ* 10,600 nm laser dosimetry and treatment protocol in achieving optimal outcomes are affirmed [33,34,47]. A prospective interventional study of 99 subjects and a case report study of medically compromised subject conducted by Hanna et al., (2016 and 2019, respectively) [33,34], as well Hanna’s chapter in a published book [47] in the management of various oral mucosal conditions and pathologies endorsed this. Also, the results of the present study consolidated the validity, efficacy and safety of this protocol.

## 5. Conclusions

Our results showed that our standardised *λ* 10,600 nm laser dosimetry protocol was effective at eradicating the persistent unresponsive PGCG with a complete resolution on the 4th day post-laser treatment, and it was sustained at two-week and 6-month follow-up timepoints with minimal to no post-operative complications, accelerating the wound healing beyond the physiological wound-healing phase. These findings could be a useful guide for healthcare professionals who utilise *λ* 10,600 nm surgical lasers for benign oral mucosal lesions management in order to choose the appropriate dosimetry and treatment protocol without comprising the integrity and safety of the surrounding hard and soft tissues. Randomised controlled clinical trials with large data comparing *λ* 10,600 nm with our dosimetry protocol to the standard surgical treatment modality at long follow-up timepoints are warranted.

## Figures and Tables

**Table 1:** Description of the utilised laser and the adjustable and calculated laser operating of carbon dioxide laser. Abbreviations: seconds (s); mL: millilitre; min: minute; mm: millimetre; J: joule; W: watt; cm^2^: square centimetre.

Device Specifications	Manufacturer	LX-20SP Nova Pulse, Lumenis, Israel
	Wavelength (nm)	10,600
	Emission mode	Gated continuous wave
	Duty cycle (%)	50% duty cycle, 5 s on/5 s off
	Delivery system	Fibre optic
	Energy distribution	Gaussian
	Beam divergence	8 degrees
Irradiation Parameters	Peak power output (W)	1.62
	Average power output (W)	0.81
	Spot diameter (mm)	0.8
	Spot diameter at focus (cm^2^)	0.005
	Spot diameter at tissue (cm)	0.1081
	Laser-to-tissue distance (mm)	<1
	Spot area at tissue (cm^2^)	0.0092
	Total energy (J)	73
	Peak fluence (J/cm^2^)	7934.78
	Peak irradiance at spot area (W/cm^2^)	324.0
	Peak irradiance at tissue (W/cm^2^)	176.08
	Average irradiance at spot area (W/cm^2^)	162
	Average irradiance at tissue (W/cm^2^)	88.04
	Speed of the movement (mm/s)	~2
Treatment Protocol	Total treatment duration (s)	90
	Water irrigation (mL/min)	12
	Air (mL/min)	20–25 (Manufacturer instructions)
	Aspirating airflow (mL/min)	300 (Manufacturer instructions)

## Data Availability

All the data are available in the text.
